# Determinants of influenza and pneumococcal vaccine uptake among preschool children in Singapore

**DOI:** 10.1371/journal.pone.0285561

**Published:** 2023-05-17

**Authors:** Marina Zahari, Vittoria Offeddu, Gavin J. D. Smith, Clarence C. Tam

**Affiliations:** 1 Saw Swee Hock School of Public Health, National University of Singapore and National University Health System, Singapore, Singapore; 2 Programme in Emerging Infectious Diseases, Duke-NUS Medical, Singapore, Singapore; 3 SingHealth Duke-NUS Global Health Institute, Singapore, Singapore; 4 Duke Global Health Institute, Duke University, Durham, NC, United States of America; 5 London School of Hygiene & Tropical Medicine, London, United Kingdom; University of Western Australia, AUSTRALIA

## Abstract

Young children are at increased risk of severe illness from influenza and pneumococcal infections. The World Health Organization (WHO) recommends vaccination with influenza and pneumococcal conjugate vaccine (PCV). However, in Singapore, vaccine uptake remains suboptimal relative to other routine childhood immunisations. Limited information exists regarding determinants of influenza and pneumococcal vaccine uptake in children. We estimated vaccine uptake and investigated factors associated with influenza and pneumococcal vaccination status by age group using data from a cohort study on acute respiratory infections in children attending preschools in Singapore. We recruited children aged two to six years at 24 participating preschools from June 2017 to July 2018. We determined the proportion of children immunised with influenza vaccine and PCV, and investigated socio-demographic factors associated with vaccine uptake using logistic regression models. Among 505 children, 77.5% were of Chinese ethnicity, and 53.1% were male. History of influenza vaccination was 27.5% of which 11.7% had been vaccinated within the past 12 months. In multivariable analyses, factors associated with influenza vaccine uptake were ‘children living in landed property’ (aOR = 2.25, 95% CI [1.07–4.67]) and ‘history of hospitalisation due to cough’ (aOR = 1.85, 95% CI [1.00–3.36]). Nearly three-quarters of participants (70.7% 95%CI: [66.6–74.5]) reported prior PCV vaccination. PCV uptake was higher for younger children. ‘Higher parental education’ (OR = 2.83, 95% CI [1.51,5.32]), ‘household income’ (OR = 1.26, 95% CI [1.08,1.48]) and ‘smokers in household’ (OR = 0.48, 95% CI [0.31,0.74]) were significantly associated with PCV uptake in univariable analyses. Only ‘smokers in household’ remained significantly associated with PCV uptake (aOR = 0.55, 95% CI [0.33,0.91]) in the adjusted model. Our results indicate that episodes of severe respiratory illness are a cue to influenza vaccination suggesting that doctors are more likely to recommend influenza vaccines to high-risk children. For PCV, our findings suggest overall greater awareness and education on the benefit of PCV vaccination is required.

## Introduction

Globally, lower respiratory infections (LRI) account for an estimated 100,000 episodes and 100 deaths each year per million children aged under five years. Influenza and pneumococcal pneumonia contribute 1.28% and 52.3% of LRI deaths, respectively [1]. Both influenza and pneumococcal vaccination can reduce disease burden [2, 3]. The World Health Organization (WHO) recommends annual influenza vaccination from six months onwards [4] and three doses of pneumococcal conjugate vaccine (PCV) for children as young as six weeks of age [5].

In Singapore, the Ministry of Health (MOH) recommends—as part of the Singapore Childhood Immunisation Schedule (CIS)—an annual inactivated influenza vaccine for children between six months and five years of age and three doses of the 13-valent pneumococcal conjugate vaccine (PCV-13) at three, five, and 12 months of age [6]. Other recommendations of the Singapore CIS include vaccinations against tuberculosis, hepatitis B, polio, *Haemophilus influenza* type b, measles, mumps, rubella, diphtheria, tetanus, and pertussis and are fully subsidised in the public health sector for children who are Singaporean citizens or Singapore Permanent Residents. In contrast, PCV and seasonal influenza vaccines were only made available free of cost in November 2020. Prior to that, Singapore citizens and Permanent Residents could compensate the costs of influenza or PCV vaccination using one of the following national financial schemes; Medisave, a medical savings scheme that was made available to pay for PCV vaccination in November 2009; the Baby Bonus scheme which includes a cash gift disbursed to parents over a period of 18 months for each child born on or after 1 January 2015; and the Child Development Account (CDA), a savings account for children up to 12 years to support educational and healthcare expenses [7]. Doses of influenza and PCV vaccines could also be purchased by means of out-of-pocket payment in case the child is not eligible for the above-mentioned subsidies. Despite the national recommendations and the expansion of the financial schemes, influenza and PCV uptake remain suboptimal. National estimates showed PCV coverage increased from 17% in 2009 to 82% in 2019 [8], but this is still lower compared with other vaccinations such as measles, polio, and diphtheria [6]. A previous study showed that influenza vaccine uptake in the last 12 months among children attending preschools is around 15% [9]. Reasons for low vaccine uptake are complex and are not limited to cost of the vaccine but include various socio-demographic factors. A previous survey of 162 parents in Singapore reports that parents of PCV-unvaccinated children were less likely to know about pneumococcal disease or PCV vaccination and were less willing to pay for vaccination out of pocket compared to parents of PCV-vaccinated children [10].

Here, we evaluated socio-demographic factors associated with influenza and PCV vaccination in children attending preschools in Singapore.

## Methods

### Study population

Baseline data from a longitudinal study of the incidence and aetiology of respiratory infections in children attending preschools in Singapore was used for regression analysis [11]. We recruited participants from 24 private preschools belonging to a single operator that provides preschool education in English and is not representative of the diversity of the preschool teaching in Singapore. Children were eligible if they were aged two to six years, attended one of the participating preschools, were attending the preschool full day, generally from 9am to 6pm, and had at least one English-speaking parent or guardian. Participant recruitment took place from June 2017 to July 2018. As part of the enrollment process, parents or guardians completed a paper-based questionnaire with socio-demographic, general health, and vaccination information.

### Vaccination status

We defined vaccine uptake as immunisation with an influenza or PCV vaccine in the past as reported by the child’s parent or guardian. Information on the vaccines, including the recommended timing, popular brand names, and the illness these vaccines aim to prevent, was stated in the questionnaire to aid recall of the respondent. Parents or guardians also indicated the approximate date the child had last received an influenza vaccine.

### Covariates

Based on the date of birth, we calculated the child’s age at the time of consent and constructed age categories from two to six years old. Due to the small number of children from minority ethnic groups, we dichotomised ethnicity into Chinese and non-Chinese (Malay, Indian and other ethnicities). We defined the highest parental education as the highest level of education reported by either parent; due to small numbers, we combined categories ‘did not attend school’ (n = 2) and ‘primary school’ (n = 12) and ‘secondary school’ (n = 32) to ‘secondary school or lower’. Other covariates included child’s sex (male, female); child-related health conditions such as asthma (yes, no), hay fever (yes, no), wheezing (yes, no), history of hospitalisation due to cough (yes, no), and household factors such as housing type (Housing Development Board (HDB) public housing, condominium, landed property), monthly household income in Singapore dollars (0–3,999; 4,000–5,999; 6,000–9,999; 10,000–14,999; and >15,000) and the number of family members living in the same household (3, 4, 5, 6, ≥7).

### Statistical analysis

We calculated vaccine uptake as the percentage of enrolled children with information on their influenza vaccine and PCV status. If the parent or guardian was unsure if the child had received an influenza vaccine or PCV, we considered the child not to be vaccinated with an influenza vaccine or PCV. Next, we performed a descriptive analysis of participant characteristics. All variables were presented as categorical. To identify socio-demographic factors independently associated with vaccine uptake, we used separate multivariable logistic regression models for history of influenza and PCV vaccination. In univariable analyses, we first assessed the association between vaccination status and other factors such as parent’s highest education, household income, housing type, child’s age, presence of smokers in the household, child’s asthma, and history of hospitalisation due to cough. In multivariable analyses, we included these variables in a regression model. Finally, we estimated crude odds ratios (OR) and adjusted odds ratios (aOR) with 95% confidence intervals (CI) to identify associations between vaccination status and explanatory variables. All statistical analyses were performed using R v.4.0.5 software [12].

### Ethics statement

The study was approved by the National University of Singapore Institutional Review Board (NUS-IRB Ref: B-16-004), Singapore. Parents or guardians provided written informed consent for enrollment of their children into the study.

## Results

### Study population

A total of 1699 children were attending one of the 24 participating preschools at the time of recruitment. The majority of the children attended the preschool full-day (93.6%). Of these, 684 children were enrolled in the study. We received 540 questionnaires, of whom 35 (6.3%) were excluded from the analysis due to unreported pneumococcal or influenza vaccine status or missing more than 50% of the questionnaire’s information. Among the remaining 505 children, 53.1% were male, and 77.5% were of Chinese ethnicity (Table 1). The percentage of children with asthma, hay fever, wheezing, and egg allergies were 3.2%, 2.4%, 13.7% and 1.6%, respectively. Sixty children (11.9%) had a history of hospitalisation due to cough. The majority of children were from households residing in public housing (63.5%), a monthly household income between S$6,000 and S$10,000 (30.5%), and one or both parents with at least a university degree (71.3%). About a quarter of children lived in a household with adults who smoked (23.8%). Compared to national population data, we recruited fewer children living in HDB flats (78.7% vs 63.5%) and more children residing in condominiums (16.0% vs 28.6%) or landed properties (5.0% vs 7.9%). The lowest (S$0- S$4000) and highest (S$ <15000) income groups were overrepresented in our study cohort while other income groups (S$4000- S$6000, S$6000- S$10000, S$10000- S$15000) were underrepresented. We could not directly compare the number of people living in one household in our study with national data since our population of interest included only families with children attending preschool full day in a limited subset of the available care arrangements in Singapore [13].

**Table 1:** Socio-demographic characteristics of enrolled children aged 2–6 years. If n is less than 505, it is stated in the table and is due to missing information in the questionnaire.

			Total	
			N %	
	Number of enrolled participants		505	
*Demographics*				
	Age at enrolment (years), n = 501			
		2	134	26.7
		3	131	26.1
		4	96	19.2
		5	89	17.8
		6	51	10.2
	Sex			
		Female	237	46.9
		Male	268	53.1
	Ethnicity, n = 502			
		Chinese	389	77.5
		Non-Chinese	113	22.5
*Health status*				
	Asthma, n = 502			
		Yes	16	3.2
		No	486	96.8
	Hay fever, n = 501			
		Yes	12	2.4
		No	489	97.6
	Wheezing, n = 501			
		Yes	69	13.7
		No	433	86.3
	Egg allergies, n = 500			
		Yes	8	1.6
		No	492	98.4
	History of hospitalisation due to cough, n = 503			
		Yes	60	11.9
		No	443	88.1
*Parent’s demographics*				
	Respondent			
		Father	126	25.0
		Mother	376	74.5
		Legal guardian	2	0.4
		Grandparent	1	0.2
	Marital status, n = 502			
		Married	484	96.4
		Unmarried	18	3.6
	Parent’s highest education, n = 499			
		Secondary school or lower	46	9.2
		Pre-university	97	19.4
		University	356	71.3
*Child’s household characteristics*				
	Housing type, n = 504			
		HDB flat	320	63.5
		Condominium	144	28.6
		Landed property	40	7.9
	Number of people in household			
		≤3	123	24.4
		4	229	45.3
		5	91	18.0
		6	32	6.3
		≥7	30	5.9
	Household monthly income (S$), n = 489			
		0–4000	68	13.9
		4000–6000	65	13.3
		6000–10000	149	30.5
		10000–15000	115	23.5
		>15000	92	18.8
	Smokers in household, n = 499			
		Yes	119	23.8
		No	380	76.2
	Pets in household, n = 501			
		Yes	107	21.4
		No	394	78.6

HDB, Housing Development Board (public housing in Singapore); PCV, pneumococcal conjugate vaccine; Pre-University includes college and polytechnics.

### Uptake of influenza and pneumococcal vaccines

**Fig 1:**
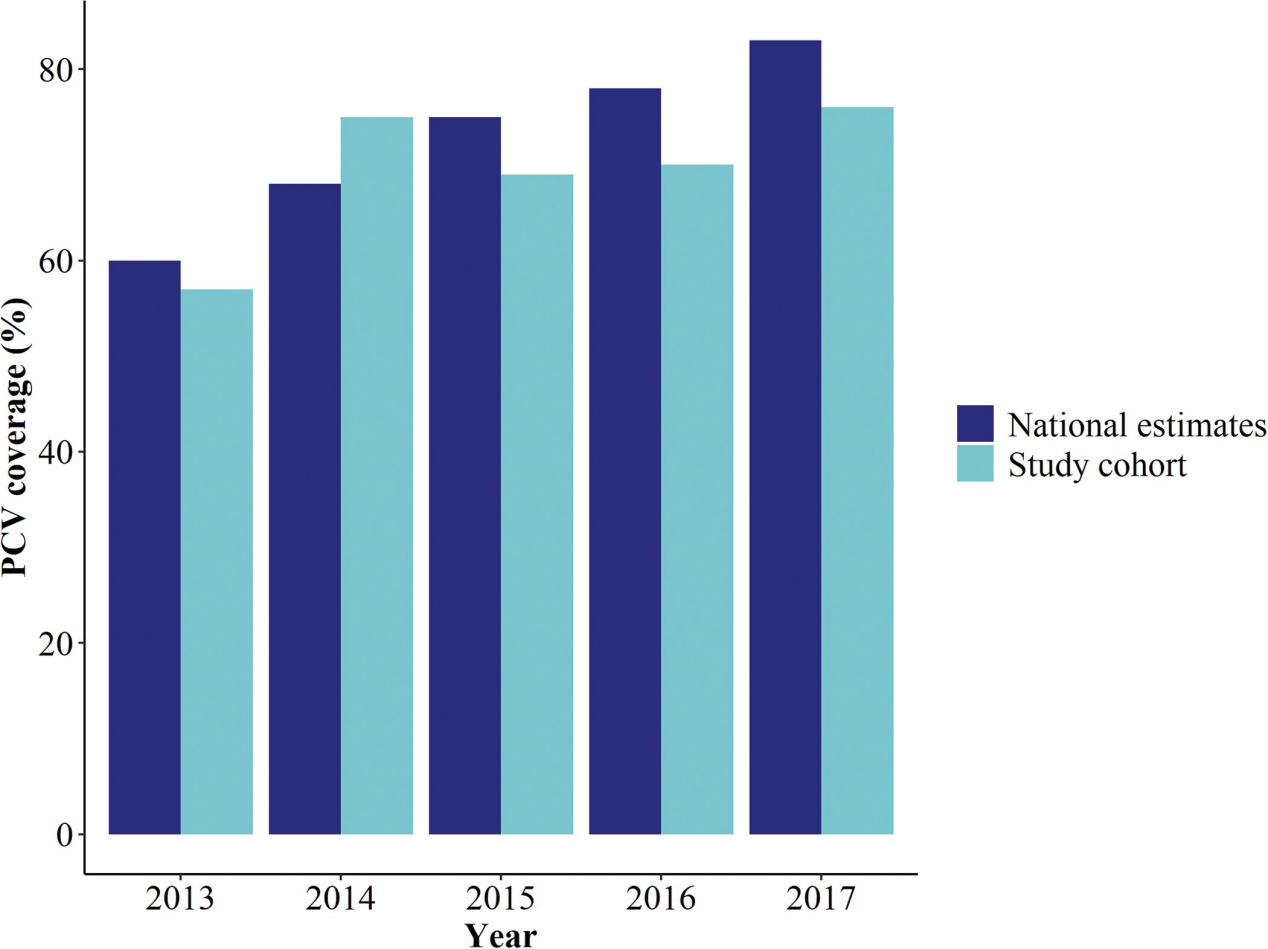
Year-specific national estimates of pneumococcal conjugate vaccine (PCV) coverage reported by the Singapore Ministry of Health (MOH) (6) (dark blue) and pneumococcal conjugate vaccine (PCV) uptake from the study cohort (light blue). National estimates are based on the percentage of infants who received the 3rd dose of PCV in a particular year. Data from the study cohort do not contain information on the number of PCV doses received, thus, estimates are based on the percentage of PCV vaccinated children (defined as immunisation with a PCV in the past, regardless of the number of doses received, as reported by child’s parent or guardian) per year of birth.

A quarter of children (27.5%, 95% CI: [23.8–31.6]) had received influenza vaccination at some point in the past; 11.7% (95% CI: [9.2–14.8]) had been vaccinated within the past 12 months. Overall, pneumococcal vaccine uptake was 70.8% (95% CI: [66.6–74.5]) and was highest in 2-year-old children (76.1%) and lowest in 6-year-old children (56.9%) (Fig 1).

### Factors associated with influenza vaccine and PCV uptake

Table 2 shows the numbers and crude and adjusted odds ratios for influenza vaccine and PCV uptake according to socio-demographic factors and history of hospitalisation due to cough. In the univariable analysis, children with history of hospitalisation due to cough were 1.77 more likely to be vaccinated with influenza compared to those never hospitalised due to cough (95% CI [1.00–3.08]). Household income, type of housing, smokers in household, and asthma were positively associated while child’s age and parental education were negatively associated with influenza vaccine uptake. However, none of these were statistically significant. In the multivariable analysis, variables that were significantly associated with influenza vaccination were type of housing (public housing vs landed property aOR = 2.25, 95% CI [1.07–4.67]) and hospitalisation (no hospitalisation vs hospitalisation due to cough aOR = 1.85, 95% CI [1.00–3.36]).

**Table 2:** Factors associated with influenza vaccine and PCV uptake in 505 preschool children (univariable and multivariable analyses). Statistically significant effect estimates are in bold.

		N	Influenza vaccine uptake			PCV uptake		
			N (%)	OR [95% CI]	aOR(95% CI)	N (%)	OR [95% CI]	aOR[95% CI]
Overall		505	139 (27.5)			357 (70.7)		
Child’s age				0.92 [0.79–1.06]*	0.91 [0.78–1.07]*		0.88 [0.76–1.02]*	0.91 [0.78–1.07]*
2			40 (28.8)			102 (28.7)		
3			40 (28.8)			91 (25.6)		
4			24 (17.3)			66 (18.6)		
5			23 (16.5)			67 (18.9)		
6			12 (8.6)			29 (8.2)		
Household monthly income (S$)				1.03 [0.88–1.20]*	0.95 [0.77–1.18]*		**1.26 [1.08–1.48]** *	1.06 [0.86–1.32]*
0–4000			19 (14.0)			39 (11.1)		
4000–6000			16 (11.8)			43 (12.3)		
6000–10000			40 (29.4)			110 (31.3)		
10000–15000			36 (26.5)			89 (25.4)		
>15000			25 (18.4)			70 (19.9)		
Parent’s highest education								
	Secondary or lower		14 (10.1)	REF	REF	24 (6.7)	REF	REF
	Pre-university		22 (15.8)	0.67 [0.31–1.49]	0.7 [0.30–1.66]	62 (17.4)	1.65 [0.81–3.37]	1.29 [0.60–2.76]
	University		102 (73.4)	0.92 [0.48–1.84]	1.01 [0.44–2.38]	269 (75.4)	**2.83 [1.51–5.32]**	2.12 [0.95–4.70]
Type of housing								
	HDB		81 (58.3)	REF	REF	225 (63.0)	REF	REF
	Condominium		41 (29.5)	1.17 [0.75–1.82]	1.11 [0.66–1.88]	101 (28.3)	0.99 [0.64–1.53]	0.71 [0.41–1.22]
	Landed property		16 (11.5)	1.97 [0.98–3.86]	**2.25 [1.07–4.67]**	31 (8.7)	1.45 [0.69–3.34]	1.42 [0.61–3.75]
Smokers in household								
	No		103 (74.1)	REF	REF	284 (79.6)	REF	REF
	Yes		34 (24.5)	1.08 [0.67–1.69]	1.02 [0.60–1.71]	70 (19.6)	**0.48 [0.31–0.74]**	**0.55 [0.33–0.91]**
Asthma								
	No		133 (95.7)	REF	REF	347 (97.2)	REF	REF
	Yes		5 (3.6)	1.21 [0.37–3.38]	1.02 [0.30–3.05]	8 (2.2)	0.40 [0.14–1.11]	0.47 [0.16–1.41]
History of hospitalisation due to cough								
	No		115 (82.7)	REF	REF	312 (87.4)	REF	REF
	Yes		23 (16.5)	**1.77 [1.00–3.08]**	**1.85 [1.00–3.36]**	42 (11.8)	0.97 [0.55–1.78]	1.11 [0.58–2.20]

N, number; OR, odds ratio; aOR, adjusted odds ratio; HDB, Housing Development Board; CI, confidence interval; PCV, pneumococcal conjugate vaccin; REF referent

* linear trend

In the univariable analysis, household income, education, and smokers in household, were significantly associated with PCV uptake. Household monthly income (OR = 1.26, 95% CI [1.08,1.48]) and highest parental education (secondary school or lower vs university; OR = 2.83, 95% CI [1.51,5.32]) were positively associated while smokers in household (smokers vs non-smokers; OR = 0.48, 95% CI [0.31,0.74]) was negatively associated with PCV uptake. In the multivariable analysis, having smokers in the household only remained significantly associated with PCV uptake (aOR = 0.55, 95% CI [0.33–0.91]), but not influenza vaccine uptake. Although households with one of the parents having at least a university degree were 2.12 times more likely to have their children vaccinated with PCV compared to households with parents without a university degree (secondary or lower), the association was not statistically significant. In the multivariable analysis, we found no significant association between household income and vaccination status for either vaccine type.

## Discussion

This study was based on data collected from a longitudinal study conducted among preschool children in Singapore. The objective was to determine socio-demographic factors associated with uptake of influenza and PCV vaccine. Our results indicate that influenza vaccine uptake in the childcare population is low, with around one in 10 children aged two to six years having been vaccinated in the previous 12 months. In contrast, pneumococcal vaccination coverage has steadily increased in more recent birth cohorts. The suboptimal uptake of influenza vaccine in preschool children in Singapore is in agreement with a previous study in which 15% of children had been vaccinated against influenza in the preceding 12 months [9]. It is lower than coverage estimates from Taiwan with reported coverage of 55% in children aged six months to three years [14]. Quantitative and qualitative studies in Singapore point to the common misconception among both parents and healthcare providers that influenza vaccination is primarily required before travelling overseas [9, 15]. This may reflect the common perception of influenza as a ‘winter disease’, despite year-round transmission in tropical settings. Furthermore, seasonal influenza vaccine composition is evaluated annually and updated if required according to the recommendations of the WHO [16]. This might make it more challenging to keep up with the annual vaccination. Furthermore, studies have shown that confusion with common terminology such as influenza, flu, and cold as well as safety concerns due to yearly influenza vaccination exist [17, 18].

Our findings indicate that children with a history of hospitalisation due to cough are more likely to be vaccinated against influenza. A previous study reported that children in Singapore were more likely to be vaccinated against influenza if recommended by the child’s doctor [9]. The important role of healthcare professionals in advising on influenza vaccination has been addressed elsewhere [19–22]. Qualitative research involving healthcare providers has shown that primary care physicians furthermore tend to recommend influenza vaccination to high risk groups such as pregnant women, the elderly, and immunocompromised [20]. Therefore, advice on the kid’s yearly influenza vaccination from healthcare providers might be lacking for children without any chronic condition or a history of severe respiratory illness.

Although previous studies have suggested that cost may be a barrier to influenza [22, 23], or pneumococcal vaccination [10], we did not find a direct association between household income and children’s vaccination status. Instead, living in landed properties was associated with increased odds of influenza vaccination in line with socioeconomic position and health-seeking behaviour, including vaccination.

Currently all vaccines included in the Singapore CIS are fully subsidised. Before November 2020, doses of influenza and pneumococcal vaccines could be obtained by means of out-of-pocket payment or by one of the national financial schemes to cover the costs, provided eligibility of the child. In our study, which was conducted in years 2017 and 2018, we saw that younger children were more likely to be vaccinated, indicating that over time, more financial support for pneumococcal vaccination led to increased PCV uptake [8].

We found that higher parental education was associated with increased odds of PCV vaccination in the univariable analysis, which may be a proxy for greater awareness of child health interventions, including immunisation. However, we did not find an association between education and influenza vaccine uptake. This could be explained by an influenza vaccine uptake of merely 27.5% (n = 139) and a relatively small sample size. Kempe et al. reported that lower ‘parental education’ and ‘parents with preschool children’ were associated with increased hesitancy for childhood vaccination and identified serious side effects following vaccination as the main concern [24].

Children living in households with smokers have an increased risk of respiratory infections contributing to the overall health care burden [25]. Therefore, it is important to understand the relationship between smokers in households and vaccine uptake in children. A study on US children aged one to 17 years reported a lower influenza vaccination rate in households with smokers [26]. However, our findings suggest lower PCV—but not influenza—uptake in children living in households with smokers. Notably, we also found that most households with smokers are those from lower-income households.

Strengths of our study are the agreement between our and previous studies conducted in similar settings, provision of additional information on factors associated with influenza and PCV vaccine uptake, and comprehensive data collection of baseline characteristics of preschool children in Singapore. However, several potential limitations should be borne in mind when interpreting the results of this study. First, information on vaccination status was parent-reported and could not be verified based on medical records leading to a potential recall bias. Nonetheless, the overall estimates are in overall agreement with official pneumococcal vaccine coverage data (Fig 1) [8]. Second, we cannot rule out the potential for social desirability bias, although this is likely to have been partly mitigated by use of a self-administered questionnaire. Third, our study was conducted among children attending private preschools only, who may not be representative of the wider paediatric population, particularly with respect to socioeconomic status. Of note, the proportion of children attending either private or public preschools in Singapore is approximately the same [27]. Finally, children attending preschool half days were not included in this study, which was only 6.4% of all children enrolled in one of the 24 preschools.

Nonetheless, our study demonstrates the need to understand factors associated with vaccination on an individual vaccine basis and to monitor these routinely to quantify the impact of vaccination policies on vaccine uptake. Earlier policy changes to minimise financial barriers for pneumococcal vaccinations may have helped to improve uptake. However, providing strategic educational messages to parents on the importance of PCV could help to reduce vaccination gaps further. In contrast to PCV, annual influenza vaccine uptake remains low and is largely reliant on opportunistic recommendations from healthcare providers. Therefore, doctors and nurses should be reminded of their crucial role in communicating the importance of influenza vaccination. Future studies will indicate whether the recent policy changes to fully subsidise influenza and PCV vaccination result in an increase in vaccine uptake.
